# Left Ventricular Sarcoma Causing Dynamic Outflow Tract Obstruction and Cardiogenic Shock: A Case Report

**DOI:** 10.1155/crcc/9317389

**Published:** 2026-01-07

**Authors:** Tomás de la Barra, Mariana Navarro, Pablo Regueira, Aníbal Zamorano, Marlon Ponce, Mario Portilla, Pablo Salazar, Tomas Regueira

**Affiliations:** ^1^ Department of Intensive Care Medicine, Clínica Santa María, Santiago, Chile, clinicasantamaria.cl; ^2^ Universidad del Desarrollo, Santiago, Chile, udd.cl; ^3^ Department of Cardiology, Clínica Santa María, Santiago, Chile, clinicasantamaria.cl; ^4^ Department of Anesthesiology, Clínica Santa María, Santiago, Chile, clinicasantamaria.cl; ^5^ Department of Cardiothoracic Surgery, Clínica Santa María, Santiago, Chile, clinicasantamaria.cl; ^6^ Universidad San Sebastián, Santiago, Chile, uss.cl

**Keywords:** cardiac sarcoma, cardiac tumor, critical care, echocardiography, hemodynamic instability, left ventricular outflow tract obstruction

## Abstract

**Background:**

Primary cardiac sarcomas are rare and aggressive tumors that often present with nonspecific symptoms, typically diagnosed at advanced stages. Obstruction of the left ventricular outflow tract (LVOT) due to these tumors is an exceptionally rare and life‐threatening complication.

**Case Presentation:**

We describe the case of a previously healthy 33‐year‐old male who presented with progressive exertional dyspnea, orthopnea, and hemoptysis. Upon admission, he quickly developed hemodynamic instability and respiratory failure. Point‐of‐care transthoracic echocardiography revealed a large intracardiac mass causing dynamic LVOT obstruction. During intubation, the patient experienced cardiac arrest, necessitating advanced cardiopulmonary resuscitation. Urgent surgical intervention confirmed the presence of an infiltrative cardiac sarcoma with positive margins. The postoperative recovery was favorable, enabling extubation and withdrawal of vasoactive agents within 24 h.

**Discussion:**

Cardiac sarcomas often mimic other cardiovascular conditions, complicating early diagnosis. Imaging techniques such as echocardiography and cardiac MRI are essential for detection and characterization. The prognosis for patients with cardiac sarcomas remains poor due to the tumors′ infiltrative nature and high rates of recurrence; however, complete surgical resection is the cornerstone of treatment.

**Conclusion:**

This case underscores the necessity for clinical vigilance in patients presenting with unexplained cardiopulmonary symptoms. The early application of bedside echocardiography facilitated prompt diagnosis and timely surgical intervention, proving life‐saving in this instance of obstructive shock due to a primary cardiac sarcoma.

## 1. Background

Primary cardiac sarcomas are a heterogeneous group of malignant tumors that originate from the connective tissues of the heart, including myocardium, endocardium, and pericardium. These tumors are rare, accounting for less than 5% of all primary cardiac tumors, and are more frequently observed in adults rather than children [1, 2]. Among these, the most common types include undifferentiated pleomorphic sarcoma, angiosarcoma, and rhabdomyosarcoma. The rarity of these tumors, coupled with their aggressive nature, often leads to delayed diagnosis and unfavorable outcomes. Patients with primary cardiac sarcomas typically present with nonspecific symptoms, such as dyspnea, chest pain, fatigue, or palpitations, which can easily be misattributed to more common cardiovascular or respiratory conditions. Due to the nonspecificity of these symptoms, many patients are diagnosed at advanced stages, which significantly affects treatment options and prognosis [3, 4].

Obstruction of the left ventricular outflow tract (LVOT) is a rare complication associated with cardiac sarcomas and can lead to life‐threatening consequences such as heart failure, syncope, or sudden cardiac arrest. Given the aggressive nature of primary cardiac sarcomas and their tendency to present with nonspecific symptoms, it is crucial for clinicians to maintain a high index of suspicion in atypical cases of cardiac distress. Early diagnosis through appropriate imaging techniques can facilitate timely intervention, potentially improving patient outcomes and survival rates in what are often life‐threatening scenarios [4, 5].

Here, we present the case of a young patient who exhibited dynamic obstruction of the LVOT and rapidly progressive dyspnea resulting from an undiagnosed cardiac sarcoma.

## 2. Case

We report the case of a 33‐year‐old male patient with no prior health issues who developed progressive exertional dyspnea over the course of 3 weeks. This was associated with hemoptysis, orthopnea, and an unquantified sensation of fever. Initially, he sought outpatient care, where he was suspected to have upper respiratory symptoms and was treated with amoxicillin‐clavulanic acid, but without improvement. Due to worsening dyspnea and orthopnea, he presented to the emergency department.

**Figure 1:**
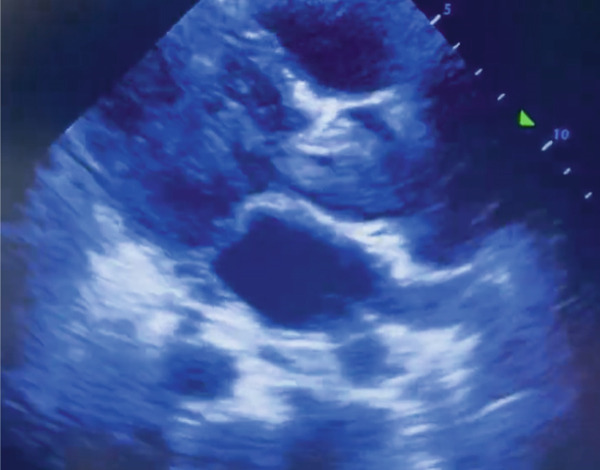
Transthoracic echocardiogram during systole demonstrating a large intracavitary mass occupying the left ventricle and extending into the left ventricular outflow tract, resulting in dynamic obstruction and severe hemodynamic compromise.

Upon admission, the patient was in stable general condition but exhibited tachycardia, tachypnea, and fever. A chest X‐ray revealed signs of pulmonary congestion. His clinical condition deteriorated, resulting in decubitus intolerance that hindered the performance of a CT scan. Laboratory tests indicated moderate microcytic anemia (Hb 8.8 g/dL), elevated C‐reactive protein (120 mg/dL), and a B‐type natriuretic peptide level of 2,800 pg/mL. Given the progressive respiratory decline, a bedside echocardiogram was conducted, which revealed a large intracardiac mass in the left ventricle causing dynamic obstruction of the outflow tract (Figure 1), along with diffuse B‐lines on lung assessment. The patient was subsequently transferred to the ICU for advanced management and further etiological investigation.

Within 2 h, the patient exhibited mild to moderate stupor, with persistent tachycardia, tachypnea, hypotension, and diaphoresis. Due to clinical deterioration and a follow‐up chest X‐ray indicating severe acute pulmonary edema, orotracheal intubation and initiation of invasive mechanical ventilation were performed. During this procedure, the patient experienced extreme bradycardia followed by asystole and a cardiorespiratory arrest, necessitating two cycles of cardiopulmonary resuscitation, which achieved restoration of spontaneous circulation. Volume resuscitation and vasoactive support with noradrenaline and vasopressin were initiated. Concurrently, both the cardiology and cardiovascular surgery teams evaluated the patient and determined that emergency surgery was required for the resection of the intracardiac mass.

**Figure 2:**
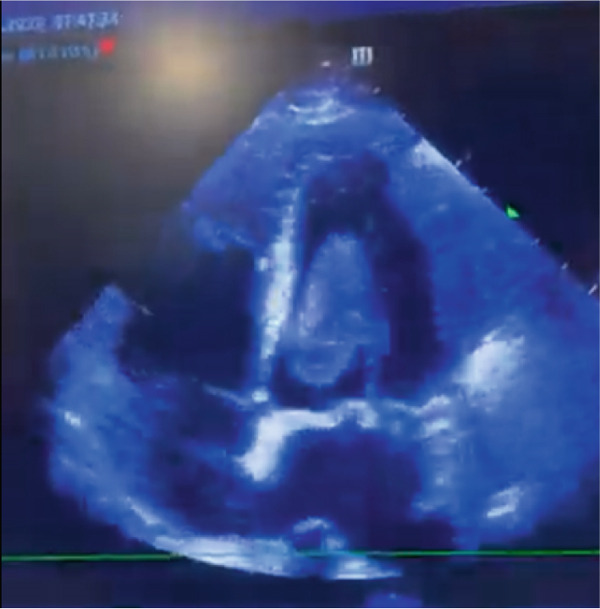
Transthoracic echocardiogram during dyastole demonstrating a large intracavitary mass occupying the left ventricle.

**Figure 3:**
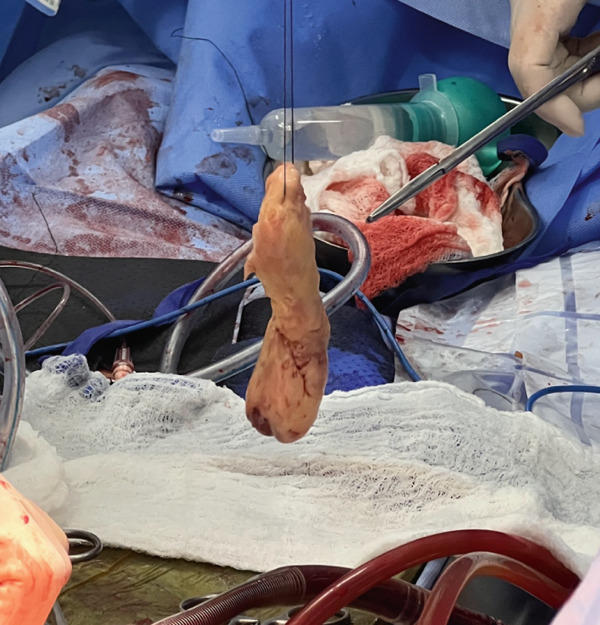
Intraoperative specimen and subsequent histopathological analysis confirming the diagnosis of an infiltrative primary intimal cardiac sarcoma, consistent with the malignant etiology of the obstructive lesion.

Intraoperatively, a rapid biopsy confirmed the diagnosis of infiltrative cardiac sarcoma with positive margins (Figures 2 and 3). Following surgery, the patient was returned to the ICU for advanced ventilatory and hemodynamic support. Over the subsequent 24 h, he showed favorable progress, achieving hemodynamic stability within a few hours, with successful withdrawal from vasoactive medications and extubation. In the following days, he was transferred to a general ward for further evaluation and management, and he was ultimately discharged home for outpatient treatment.

Final biopsy reveals a Grade 3 intimal sarcoma (FNCLCC), with 20% necrosis and a mitotic index of 10 mitoses per 10 high‐power fields (HPF). Immunohistochemistry shows positivity for FLI1, CD31, MDM2, and CDK4, and negativity for SOX10, myogenin, and desmin.

## 3. Discussion

Cardiac sarcomas are rare and aggressive primary malignant tumors of the heart, with an incidence ranging from 0.001% to 0.1%. Although they constitute a small fraction of all cardiac tumors—most of which are benign—cardiac sarcomas represent the most common primary malignant neoplasm of the heart [6–8]. These tumors most frequently arise in the right atrium (40%–45%), predominantly due to angiosarcoma, which is the most common cardiac sarcoma overall. The left atrium is the next most common site (25%–30%), where undifferentiated pleomorphic sarcoma and intimal sarcoma predominate. Less frequently, they involve the right and left ventricles (10%–15% each), typically as rhabdomyosarcoma, angiosarcoma, leiomyosarcoma, or undifferentiated sarcoma. The pericardium is affected in fewer than 10% of cases.

Intimal sarcoma, as in the present case, arises from the intimal layer of large vessels, most commonly the pulmonary artery, aorta, or pulmonary veins. In this patient, the tumor most likely originated from the aortic intima, extending into the left atrium and left ventricle [9–12].

Although LVOT obstruction is classically associated with hypertrophic cardiomyopathy [13] or valvular and subvalvular disease among others [14], there are rare documented instances in which cardiac sarcomas have produced dynamic or nearly fixed LVOT obstruction via intracavitary tumors. For example, Rocco et al. described the first reported case of acute critical LVOT obstruction caused by a cardiac myxoid spindle cell sarcoma, highlighting how the mass projected into the outflow tract and generated a significant pressure gradient [15]. Similarly, Grimaldo et al. reported a 57‐year‐old man with high‐grade synovial sarcoma involving the left ventricle, where the intracardiac tumor induced dynamic obstruction of the LVOT, mimicking subaortic obstruction [16]. In another case, an intimal (spindle cell) sarcoma was observed to produce both LVOT obstruction and functional mitral stenosis, underscoring that aggressive sarcomas can be involved on multiple intracardiac flow pathways simultaneously [17].

Clinical manifestations of cardiac tumors depend on their location, size, and hemodynamic effects. Common symptoms include intracardiac obstruction, systemic embolization, and nonspecific symptoms such as fever and weight loss [12–14]. Benign tumors, such as myxomas, primarily present with signs of obstruction or embolization, with an estimated 30% to 40% potentially leading to ischemic stroke [18]. In contrast, malignant tumors, including cardiac sarcomas, can infiltrate myocardial tissue, resulting in severe arrhythmias and heart failure. They may also lead to sudden cardiac death due to arrhythmias or embolic events [19, 20].

The diagnosis of cardiac sarcomas is challenging due to their low incidence and the overlapping clinical symptoms with other cardiovascular conditions. Advanced imaging techniques are crucial for the early detection and characterization of these tumors [20]. Echocardiography is typically the first imaging modality utilized, valued for its accessibility and ability to assess tumor location, size, and mobility. Transesophageal echocardiography (TEE) further enhances evaluation, particularly of structures in the posterior heart [21, 22].

Cardiac magnetic resonance (CMR) imaging is essential for detailed tissue characterization and often reveals masses that may be missed by echocardiography. CMR provides critical information regarding tumor histopathology, with specific findings such as T2 hyperintensity and late contrast uptake indicative of malignancy. It also aids in determining the extent of the tumor and its relationship to cardiac and extracardiac structures, which is vital for treatment planning [23].

Computed tomography (CT) and positron emission tomography (PET) may be employed, particularly for patients who are unable to undergo CMR. When combined with CT or CMR, PET offers metabolic characterization that helps differentiate between benign and malignant lesions [24]. In some cases, a transvenous biopsy may be performed to confirm the histological diagnosis, especially if malignancy is suspected based on imaging. However, a multimodal imaging approach is often sufficient for diagnosis without necessitating a biopsy in many instances [25].

Management of cardiac sarcomas is complex and requires a multidisciplinary approach, integrating surgery, chemotherapy, and in select cases, radiotherapy. Despite aggressive treatment strategies, the prognosis remains poor, with median survival generally ranging from 6 to 12 months. Complete surgical resection with negative margins (R0) is the most significant prognostic factor that can enhance survival, although achieving this is often difficult due to the infiltrative nature of these tumors [5, 13].

Adjuvant chemotherapy utilizing regimens such as anthracyclines (e.g., doxorubicin) and alkylating agents (e.g., ifosfamide) is commonly employed to optimize postoperative outcomes, showing some benefit in patients with cardiac sarcomas. Paclitaxel has been used in certain cases; however, its efficacy remains limited and requires further investigation [9]. Radiotherapy can serve as a complementary treatment, particularly for inoperable patients, and may enhance progression‐free survival [26].

Overall, the prognosis for cardiac sarcomas is unfavorable, with median survival ranging from 9.6 to 16.5 months. Nevertheless, multimodal therapeutic strategies have extended survival in some cases up to 27 months. The presence of metastases at the time of diagnosis is a negative prognostic factor that significantly impacts life expectancy [27].

## 4. Conclusion

Cardiac sarcomas are rare and aggressive tumors that present significant challenges for early diagnosis due to their nonspecific clinical manifestations. In the case presented, the patient experienced progressive dyspnea and orthopnea, which ultimately culminated in obstructive shock caused by a tumor obstructing the LVOT. This situation underscores the critical need for prompt diagnosis and intervention. Bedside echocardiography played a vital role in the early detection and management of this condition. Although advanced imaging techniques may facilitate tumor characterization, late‐stage diagnosis often restricts treatment options and adversely affects prognosis. Surgical resection with negative margins is essential for improving survival; however, achieving this is rarely possible due to the infiltrative nature of these tumors. Overall, the prognosis for patients with cardiac sarcomas remains poor, with a median survival of less than 1 year, as tumor recurrence and metastasis significantly impede long‐term cure. This case highlights the importance of clinical vigilance, early diagnosis, and immediate therapeutic action to enhance patient outcomes in this devastating condition.

## Consent

Written informed consent was obtained from the patient for publication of this case and any accompanying images or clinical information.

## Disclosure

The authors reviewed and edited the content as needed and take full responsibility for the content of the publication.

## Conflicts of Interest

The authors declare no conflicts of interest.

## Funding

No funding was received for this manuscript.

## Data Availability

The data that support the findings of this study are available on request from the corresponding author. The data are not publicly available due to privacy or ethical restrictions.
